# Investigation of the Use of Artificial Intelligence in Anterior Loop Detection: A Panoramic Radiography Study

**DOI:** 10.3390/diagnostics16142213

**Published:** 2026-07-15

**Authors:** Ezgi Uzun, Derya İçöz, Burak Kerem Apaydın, Kaan Orhan

**Affiliations:** 1 Department of Oral and Maxillofacial Radiology, Faculty of Dentistry, Pamukkale University, Denizli 20160, Türkiye; euzun@pau.edu.tr; 2 Department of Oral and Maxillofacial Radiology, Faculty of Dentistry, Selcuk University, Konya 42100, Türkiye; deryayilmaz@selcuk.edu.tr; 3 Department of Oral and Maxillofacial Radiology, Faculty of Dentistry, Ankara University, Ankara 06500, Türkiye; kaan.orhan@dentistry.ankara.edu.tr; 4 Medical Design Application and Research Center (MEDITAM), Ankara University, Ankara 06500, Türkiye; 5 Department of Oral Radiology, School and Hospital of Stomatology, Cheelo College of Medicine, Shandong University, Jinan 250012, China

**Keywords:** anterior loop, artificial intelligence, deep learning, mandibular canal, panoramic radiography

## Abstract

**Background/Objectives:** The accurate detection of the anterior loop (AL) of the inferior alveolar nerve is critical to avoid neurosensory complications during surgical procedures in the interforaminal region, and panoramic radiography continues to be widely used in routine dental diagnostics due to its accessibility and cost-effectiveness. This study aimed to evaluate the performance of a deep learning approach in automatic detection of the AL in panoramic radiographs. **Methods:** A total of 305 anonymised panoramic radiographs containing 413 annotated ALs were used to train a YOLOv8x-based model for automatic AL detection. The dataset was divided into training, validation, and test sets consisting of 245 images (332 AL annotations), 30 images (40 AL annotations), and 30 images (41 AL annotations). Labelling was carried out by using the polygonal annotation method. The model’s performance in identifying the AL region was measured using precision, recall, F1 score, and mean average precision (mAP@0.5). **Results:** The model achieved a precision of 0.75, a recall of 0.6585, and a F1 score of 0.7013. The average precision at an intersection over union (IoU) threshold of 0.5 (mAP@0.5) was 0.739. **Conclusions:** This study demonstrates the feasibility of using a YOLOv8x-based detection model to detect ALs in panoramic radiographs. Although further improvements are needed to enhance model sensitivity and generalisability, the findings demonstrate the potential to support clinical decision-making.

## 1. Introduction

One of the most frequently observed anatomical variations in the inferior alveolar nerve is known as the anterior loop (AL) [1]. The AL is defined as the course of the nerve extending anteriorly before reaching the mental foramen and then turning superiorly and posteriorly to exit through the foramen [1,2,3]. This variation requires particular attention during surgical procedures such as implant placement, endodontic treatment, periapical surgery, osteotomy, and genioplasty in the anterior region of the mental foramen to prevent nerve injury [1,4,5,6]. Failure to identify the presence of AL may result in nerve damage, potentially leading to sensory disturbances such as paraesthesia, hypoesthesia, or anaesthesia [1,7]. Therefore, detection of the AL is extremely important prior to performing surgical interventions in the interforaminal region [1,5,6,7].

Various imaging modalities are utilised in clinical practice to detect AL, with panoramic radiography and cone-beam computed tomography (CBCT) being the most commonly used methods for preoperative evaluation [8]. Panoramic radiography has limitations such as superimposition, distortion, and magnification, which hinder the accurate assessment of structures such as the AL located in the interforaminal region [2,3,8]. Additionally, the AL may be difficult to distinguish on panoramic radiographs due to its intermedullary localization and its position within an area surrounded by relatively thick cortical bone structures [9]. In contrast, CBCT provides more reliable diagnostic information for the evaluation of the AL owing to its three-dimensional (3D) imaging capability without superimposition or distortion; however, it also has certain disadvantages, including a higher radiation dose, artefacts related to metallic restorations, higher cost, and longer scanning time [8,9,10]. A recent CBCT study demonstrated that mandibular anatomical variations, including changes in mandibular angulation, significantly affect preoperative assessment and planning with regard to implant placement in the premolar region, and these findings highlight the necessity for careful and precise CBCT evaluation [11]. Additionally, the operator’s knowledge and experience play a crucial role in the analysis and interpretation of 3D radiographic data [12]. However, distinguishing small and complex anatomical variations, such as the AL of the mandibular canal, can be challenging even for experienced clinicians [6].

In this context, the utilisation of artificial intelligence (AI)-based approaches in the evaluation of anatomical structures is becoming increasingly prevalent, with the objective of enhancing the diagnostic performance of human observers [6,12,13]. AI-based methods offer the potential to accelerate data recognition and enhance diagnostic accuracy in image interpretation while also eliminating errors caused by human fatigue and subjective approaches [13,14]. In particular, deep learning algorithms, a subfield of AI, stand out in image analysis because of their multilayered structure and automatic feature extraction capabilities [15]. These algorithms are widely used in the healthcare field, and deep convolutional neural networks (CNNs) play a significant role in this area. CNNs are neural networks with multilayered sensors and a feedforward architecture [12,16,17]. These networks, which are highly successful in analysing image data, offer significant advantages over traditional methods in terms of accuracy, time efficiency, and consistency and are effectively utilised in tasks such as detection, quantification, classification, and segmentation [18,19].

Deep learning architectures have been utilised in various areas in dentistry such as tooth classification [16], caries detection [15], dental fillings [18], vertical root fracture examination [20], oral lesion analysis and malignancy diagnosis [21] detection of the mandibular canal [12,22,23], and evaluation of anatomical structures such as the incisive canal [19]. Previous studies have demonstrated that deep learning models can achieve high diagnostic performance in terms of identifying anatomical structures and can support clinical applications such as implant planning [19,22,23,24,25]. However, these studies focus on the detection of more clearly defined anatomical structures such as the mandibular canal and the mental foramen [22,23,24,25]. In contrast, studies addressing more complex anatomical variations such as the AL remain limited [6].

In this context, this study aims to evaluate the performance and effectiveness of the YOLOv8x architecture, a deep learning algorithm, in detecting the AL on panoramic radiographs. Since accurate detection of the AL is crucial in surgical treatments, this study aims to contribute to AL detection processes on panoramic radiographs through deep learning.

## 2. Materials and Methods

### 2.1. Study Design

**Figure 1:**
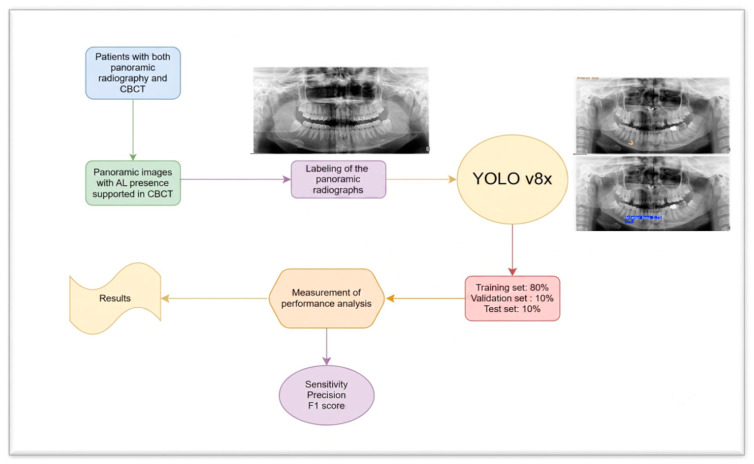
Workflow diagram of the study. The panoramic radiographs on the right show a representative example of AL detection. The upper image shows the ground-truth annotation, whereas the lower image shows the AI model detection.

This study was conducted following ethical principles and approved by the Ethics Committee of Pamukkale University (Approval No: E-60116787-020-481637, Date:23 January 2024). All procedures involving human participants complied with the ethical standards of institutional and national research committees and with the Helsinki Declaration on ethical standards. The manuscript for this study was prepared in accordance with the Checklist for Artificial Intelligence in Medical Imaging (CLAIM) and the Standards for the Reporting of Diagnostic Accuracy Studies (STARD) guidelines. In the present study, a YOLOv8x model implemented in PyTorch version 2.0 (CranioCatch, Eskisehir, Türkiye) was used to create a model for AL detection in panoramic radiographs (Figure 1).

### 2.2. Image Selection and Radiographic Dataset

**Figure 2:**
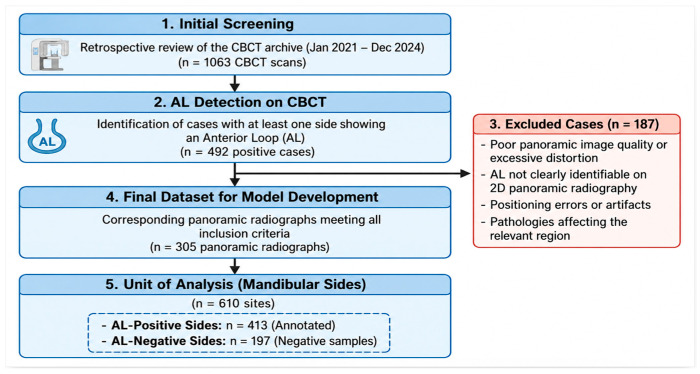
Flow diagram of the study according to STARD guidelines, illustrating the selection process from initial CBCT screening to final dataset construction, including AL-positive and AL-negative cases.

In this study, the records of patients who underwent both panoramic radiography and CBCT at the Pamukkale University Faculty of Dentistry between January 2021 and December 2024 were retrospectively evaluated. Initially the presence of the AL was determined using CBCT images as the reference standard. Patients with AL detected on at least one mandibular side on CBCT were included in the study, while cases with no AL observed on either side were excluded. Subsequently, the corresponding panoramic radiographs of the included cases were reviewed, and images meeting the inclusion criteria and considered suitable for AL annotation were selected. The final dataset consisted of 305 panoramic radiographs from patients with CBCT-confirmed AL presence. Since AL may occur unilaterally or bilaterally, a total of 413 AL annotations were obtained from these images. The contralateral sides without AL were considered within-patient negative references. Accordingly, a total of 610 mandibular sides (413 positive, 197 negative) were included in the analysis (Figure 2).

The dataset was split into training, validation, and test sets at the patient level, specifically taking bilateral annotations into account. In this way, it was ensured that images from the same patient were not included in different subsets.

All panoramic images were obtained with the use of the same device (Instrumentarium OP200D, Tuusula, Finland) with scanning parameters of 60 kV, 6.3 mA and a 14.1 s exposure time. Panoramic images with adequate diagnostic quality, free from artefacts or pathologies affecting the relevant region and obtained from individuals over the age of 18 were included in the dataset. The images containing severe technical errors that would negatively affect radiological interpretation were not included in the dataset.

### 2.3. Ground-Truth Labelling

Labelling is the procedure of identifying objects in an image and determining the region to which they belong [13]. In this study, ground-truth annotations were performed using CranioCatch software (Version 2.1; CranioCatch, Eskisehir, Türkiye), a CE-certified AI-assisted annotation platform specifically developed for use in dental radiology. CranioCatch is a platform that provides advanced image processing, polygonal annotation, and integration with PyTorch-based models, enabling the development of diagnostic tools for oral and maxillofacial radiology [26]. All annotations were performed according to predefined standard criteria by an oral and maxillofacial radiologist with 10 years’ experience. The visible boundaries of each AL were manually delineated using the polygonal annotation tool. In cases where the anatomical boundaries of the AL could not be clearly identified, the annotations were jointly reviewed with a second experienced oral and maxillofacial radiologist, and the final annotation was established by consensus. The finalized annotations were used as the ground truth for model training and evaluation.

### 2.4. Model Pipeline and Training Phase

In the present study, an AI algorithm (Cranio-Catch, Eskisehir, Türkiye) was developed for the automatic detection of AL. The model training was conducted at the Osmangazi University Faculty of Dentistry, Dental-AI Laboratory, utilising a high-performance computing infrastructure. The computational environment consisted of a Dell PowerEdge T640 Calculation Server, a Dell PowerEdge T640 GPU Calculation Server, and a Dell PowerEdge R540 Storage Server (Dell Inc., Austin, TX, USA). A total of 305 panoramic radiographs and 413 segmentation labels were used in this study. The dataset was randomly divided into training (80%), validation (10%), and test (10%) subsets. The images were initially standardised to 640 × 320 pixels for dataset management; subsequently, during the training phase, the YOLOv8x model utilized an input resolution of 1280 pixels to optimize feature extraction across the network layers.

Model training was conducted via the YOLOv8x segmentation architecture for a total of 700 epochs. This epoch limit was selected to enable the extra-large network architecture to fully learn the complex features of a highly variable anatomical structure like the AL and to achieve complete model convergence. To mitigate the risk of overfitting during the training process, a model checkpointing system was implemented, preserving only the weights that yielded the highest mean average precision (mAP@0.5) on the validation dataset. Consequently, the final model was selected based on the peak validation performance rather than the parameter weights of the final training epoch. The training process was performed using PyTorch as the backend, with additional support from libraries including OpenCV, NumPy, Pandas, TorchVision, TensorBoard, and Seaborn 0.3x for data handling, visualisation, and monitoring purposes.

Although the YOLOv8x-seg architecture was employed, the critical factor in evaluating the AL in clinical applications was not pixel-level segmentation but the reliable spatial localization of the anatomical structure. Therefore, the model outputs were treated as bounding-box-based detections. Model training was optimized using a multi-task loss function comprising Complete Intersection over Union (CIoU) and Distribution Focal Loss (DFL) for bounding box regression, and Binary Cross-Entropy (BCE) loss for classification and mask prediction. Standard detection metrics (precision, recall, F1 score, and mAP@0.5) were utilised for performance evaluation, and the results were presented as bounding boxes (Table 1).

To enhance model robustness and generalisability, extensive data augmentation was applied. Image-level augmentations were implemented via the Albumentations library, including Blur (*p* = 0.05), CLAHE (clip_limit = 2, *p* = 0.05), GaussNoise (*p* = 0.05), ISONoise (*p* = 0.1), MultiplicativeNoise (*p* = 0.1), RandomBrightnessContrast (*p* = 0.05), RandomSnow (*p* = 0.05), Sharpen (*p* = 0.05), and ToSepia (*p* = 0.005).

In addition, the following YOLO-native augmentations were employed: HSV-Hue, hue-saturation, HSV-value, and mosaic transformations.

Model performance was monitored during training via the use of TensorBoard, with key evaluation metrics including the mean average precision (mAP), intersection over union (IoU), precision, and recall scores calculated on the validation and test sets.

### 2.5. Performance Metrics

To evaluate the performance of the model, several evaluation metrics and procedures were applied. A confusion matrix, which provides a comparison between the model’s predictions and the actual labels, was constructed as a foundation for performance analysis. In this matrix, true positives (TPs) represent instances in which the model correctly detected the presence of the AL. False positives (FPs) are instances in which the model detected AL when it was not present, and false negatives (FNs) correspond to cases where the model failed to detect AL when it was present.

Model predictions were evaluated using a confidence threshold of 0.25. Non-Maximum Suppression (NMS) was applied with an IoU threshold of 0.7.

Based of these values, the following performance metrics were computed:

- Sensitivity (Recall): TP/(TP + FN)
- Precision: TP/(TP + FP)
- F1 score: 2TP/(2TP + FP + FN)

Additionally, the mean average precision (mAP) at an intersection over an IoU threshold of 0.5 (mAP@0.5) was calculated to further quantify the model’s accuracy in terms of object detection. The mAP@0.5 metric evaluates the average precision across all detected instances, considering a detection as correct when the IoU between the predicted and actual bounding boxes is at least 0.5. This value reflects both localisation and classification performance in a single score, with values closer to 1 indicating a higher degree of accuracy. Furthermore, in accordance with the Standards for Reporting of Diagnostic Accuracy Studies (STARD) guidelines, 95% confidence intervals (CIs) were calculated for the primary performance metrics. The 95% CIs for precision and recall were estimated using the exact Clopper–Pearson method, whereas the 95% CIs for the F1-score and mAP@0.5 were estimated using non-parametric bootstrap resampling at the patient level. All analyses were performed using Python 3.10 (Ultralytics YOLO, NumPy, and SciPy).

## 3. Results

A total of 305 panoramic radiographs with 413 AL annotations were included in the study. The dataset was split into 245 training images (332 labels), 30 validation images (40 labels), and 30 test images (41 labels). The YOLOv8x segmentation model was trained over 700 epochs with a learning rate of 0.01 and an input resolution of 1280 pixels (imgsz = 1280) to optimize spatial feature extraction during the training process.

**Figure 3:**
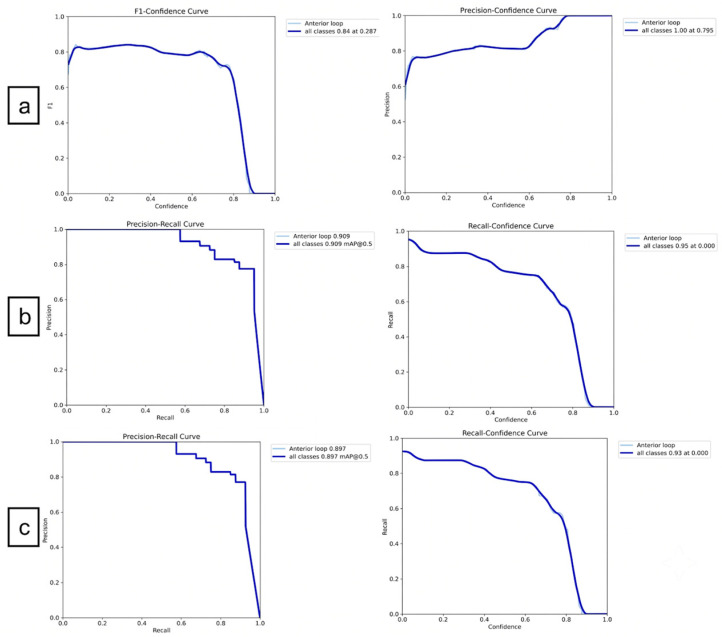
Performance curves of the YOLOv8x model on the test dataset. (**a**) F1 score vs. confidence threshold; (**b**) precision and recall vs. confidence threshold; (**c**) precision–recall curve.

Figure 3 illustrates the model’s performance variation across different confidence thresholds for AL detection by presenting the F1–confidence, precision–confidence, precision–recall, and recall–confidence curves obtained after training. The graphs demonstrate that the model maintains stable precision and F1 scores, even at high confidence levels, whereas the recall tends to decrease as the degree of confidence increases. These findings indicate that the model performs reliably in detecting AL and provides more consistent results at higher confidence thresholds.

**Figure 4:**
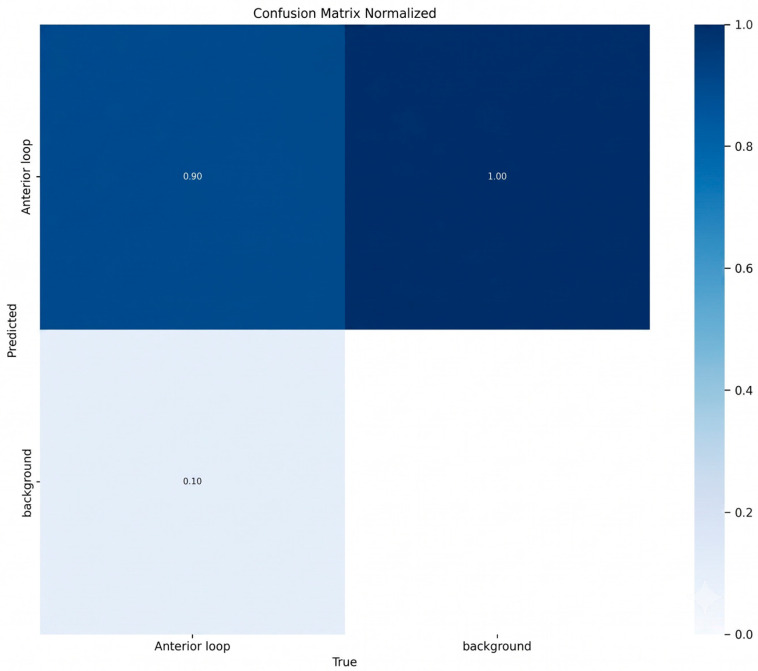
Normalized confusion matrix of the YOLOv8x model on the test dataset for AL detection.

Figure 4 illustrates the normalized confusion matrix and presents the class-level proportional prediction distribution. While this visualization reflects overall class-level performance, the metrics reported in Table 2 were calculated at the instance level using outputs obtained at the selected confidence threshold. Therefore, minor discrepancies may have arisen between the visual and numerical evaluations. According to Figure 4, the model correctly classified the AL class in 90% of cases, while misclassifying it as background in 10% of cases; the background class was classified with 100% accuracy. These results indicate that the model demonstrates moderate sensitivity in detecting AL, although some instances are still confused with the background.

**Figure 5:**
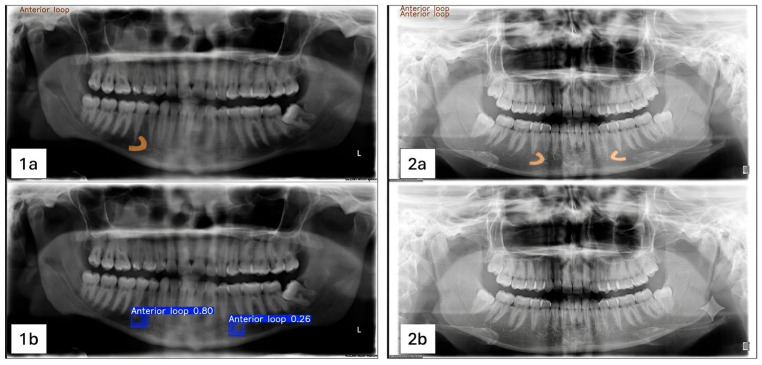
Representative examples of AL localization and segmentation by the YOLOv8x model. The images illustrate the model’s performance in delineating the anatomical boundaries of the AL using polygonal segmentation masks. (**1a**,**1b**) An image showing a true positive on the right side and a false positive on the left side. (**2a**,**2b**) A case showing false negatives on both sides. Orange-colored areas (**1a**,**2a**) represent the ground-truth polygonal annotations. Blue bounding boxes (**1b**) represent the model’s automated detections with associated confidence scores.

Polygonal segmentation masks are shown in Figure 5, illustrating the high degree of boundary alignment, although the final evaluation was performed based on localization (detection) metrics.

Table 2 summarizes the final performance results at the selected confidence threshold. Accordingly, the model achieved a precision of 0.75 (95% CI: 0.59–0.86), a recall of 0.6585 (95% CI: 0.51–0.78), and an F1-score of 0.7013 (95% CI: 0.58–0.80). In addition, the mAP@0.5 was 0.739, and the mAP@0.5:0.95 was 0.297. These metrics were derived from 27 TPs, 9 FPs, and 14 FNs obtained from the test set.

## 4. Discussion

Although the mandibular intermental region is generally regarded as a safe site for various surgical procedures, a precise understanding of its anatomical structures is crucial for preventing damage to the neurovascular bundle [3,27]. One of the most clinically significant anatomical variations in this region is the AL of the inferior alveolar nerve, the oversight of which may result in advertent nerve injury, sensory deficits or other complications [2,3]. In a prevalence study conducted by Raju et al. [3] using CBCT images, the presence of AL was detected in 25% of patients and 24% of hemisections. In another study by Vujanovic-Eskenazi et al. [2], the presence of AL was investigated in panoramic radiography and CBCT images, and the prevalence was determined as 36.6% in panoramic radiographs and 48.8% in CBCT images. Due to its significant anatomical variability and the potential for surgical complications, the presence of the AL should be meticulously assessed before procedures in the interforaminal region.

AI-driven methodologies, particularly deep learning–based models, have emerged as promising tools in diagnostic radiology, showing substantial improvements in detecting complex features with a high degree of accuracy [13,17,28,29]. Building upon these technological advancements, in this study, by accepting the presence of AL confirmed on CBCT as the reference standard, we retrospectively annotated panoramic images and trained a deep learning model to recognize this anatomical variation. In this context, a total of 305 panoramic radiographs and 413 annotated ALs were used to train a YOLOv8x architecture. Since a single panoramic radiograph could contain more than one AL, each AL identified on the right and/or left side of the mandible was labeled as a separate instance.

The YOLO architecture reframes object detection as a single regression problem, estimating bounding boxes and class probabilities directly from full images in a single imputation, eliminating the need for complex multistage detection pipelines such as Region-Based Convolutional Neural Networks (R-CNNs) or Deformable Part-Based Models (DPMs). In addition to providing a speedy analysis, YOLO encodes contextual information by processing the entire image during training and inference, reducing false positives compared with region proposal-based methods [30]. Several studies in dental radiology have utilised the YOLO architecture, reporting encouraging outcomes in tasks such as anatomical structure detection and dental disease diagnosis [18,31,32,33]. In a study conducted by Bayrakdar et al. using the YOLOv8 architecture, the detection and segmentation of 33 different conditions including dental problems, restorations, implants and anatomical structures in panoramic radiographs, were successfully performed with a high degree of accuracy (0.99–1), sensitivity and F1 scores [34]. In another study utilising the YOLOv5 architecture for the detection of fillings and overhanging fillings, the F1-score was reported to be 0.96 for fillings and 0.87 for overhanging fillings [18]. These findings highlight the versatility and strong diagnostic potential of the YOLO architecture in dental radiology.

In this study, the created model demonstrated strong overall performance by achieving a precision of 0.75 and a recall of 0.6585, and produced an F1 score of 0.7013. These metrics reflect a balanced ability to detect AL. In particular, the average precision was 0.739 at an IoU threshold of 0.5 (mAP@0.5), demonstrating the robustness of the model in detecting anatomically small and variable features such as AL. To the best of our knowledge, no prior study has specifically evaluated the automatic detection or segmentation of the AL on panoramic radiographs using deep learning methods. This study is the first to demonstrate that AI can be applied to panoramic images to automatically identify AL, providing a reproducible and objective assessment. While previous studies have primarily focused on the manual identification of AL using panoramic radiographs or CBCT, or employed AI on CBCT datasets for AL detection, our approach introduces automation directly with regard to panoramic images. In a study evaluating the automatic detection and segmentation of the inferior alveolar nerve in panoramic radiography images, a Dice score of 0.847 was reported. This shows the encouraging results of deep learning-based approaches in the detection of anatomical structures in support of clinical decision-making processes [34]. This high degree of accuracy reflects the benefits of learning 2D images, as panoramic radiographs have a higher resolution compared to CBCT, where the canal occupies a larger portion of the overall image. However, panoramic radiographs are limited in their ability to accurately depict the true three-dimensional (3D) morphology of complex canal structures [35].

In a study by Oliveira-Santos et al. [6], a cloud-based AI tool trained on 3D CBCT scans for the segmentation of the mandibular canal and AL reported substantially higher recall and accuracy values of 0.961 and 0.998, respectively. The discrepancy in these performance metrics compared to our results can be attributed to the inherent differences between imaging modalities; while their model operated on high-resolution 3D data, our study utilized two-dimensional (2D) panoramic radiographs, which involve inherent challenges such as anatomical superimposition and distortion. Despite these 2D-related difficulties, our precision (0.75) and F1-score (0.7013) values remain competitive, and are even higher in terms of precision than those reported in the aforementioned CBCT study (0.677). This suggests that our YOLOv8x-based approach involving panoramic images provides a more conservative and reliable detection with fewer false positives compared to their segmentation model, highlighting the clinical feasibility of AI-assisted AL detection on the most widely accessible imaging modality in routine dental practice.

In addition to the abovementioned factors affecting the accuracy of deep learning approaches, different deep learning networks may also yield different automatic segmentation and detection performance results. Kwak et al. [35] analysed the performance of three different deep learning networks, 2D and 3D U-Nets and 2D SegNet, for the detection and segmentation of the mandibular canal in CBCT images. Among them, 3D U-Net achieved the highest accuracy level of 0.99 [35]. It can be observed that the performance of deep learning-based anatomical structure detection is influenced by multiple factors, including the imaging modality, the architecture of the deep learning model, and the specific characteristics of the dataset used.

The relatively small size of the dataset used in our study is a recognized limitation that may increase the risk of overfitting. To address this, validation-based monitoring was employed, and extensive data augmentation was applied to increase the variability of the training data. While k-fold cross-validation could provide additional robustness, the current approach utilizing an independent test set allowed the model to achieve a moderate-to-high degree of performance, suggesting that overfitting was mitigated to a reasonable extent. Future studies with larger, multi-center datasets are warranted to further enhance model generalisability.

In this study, it was decided to obtain the images from a single panoramic radiography device with fixed acquisition parameters from a single center to ensure sample homogeneity. However, the single-center design, the use of only one panoramic imaging system, and the relatively small sample size are factors that limit the generalizability of the model. In addition, the dataset used in this study comprised CBCT-confirmed AL regions as positive samples, whereas negative samples were obtained from the contralateral sides of the same patients where no AL was identified. Although this approach provided a reliable reference standard, it may not fully reflect the anatomical diversity encountered in routine clinical practice. Therefore, in order for the model to be considered reliably applicable across different populations, external validation studies using independent datasets obtained from multiple centers and different imaging systems are required. Another limitation is that AL annotations were primarily performed by a single radiologist, which may have introduced subjective bias. In unclear cases, a second radiologist reviewed the images and consensus decisions were arrived at, but future studies could benefit from multi-observer annotation and formal inter-observer reliability assessment. Additionally, the use of two-dimensional imaging may have caused difficulties in detecting AL, especially given anatomical variations such as a less cortical mandibular canal in the anterior region and narrowing in diameter. The use of 3D imaging, multiple observers, and multiple devices in future studies may improve accuracy in evaluating delicate anatomical structures such as AL.

In addition to methodological and performance considerations, it is important to discuss the potential clinical applicability of the proposed AI model. Performing AL detection on panoramic radiographs based on deep learning could serve as a decision support tool for dentists during preoperative implant planning by highlighting areas that may require further evaluation. This could help to reduce the risk of unwanted nerve damage and improve patient safety. While the current study focuses on the development and validation of the algorithm as a proof of concept, future studies could include the integration of such AI models into user-friendly clinical software platforms, providing real-time assistance and workflow support in dental practice. Furthermore, the annotations used to train and evaluate the YOLOv8x model were performed using the CranioCatch platform, which ensures standardised and reproducible labelling of anatomical structures. Leveraging this platform adds reliability to the dataset and highlights the potential for integrating the trained model into existing AI-assisted dental imaging systems, thereby facilitating clinical translation and practical implementation in daily dental workflows.

## 5. Conclusions

This study demonstrated the feasibility of a YOLOv8x-based deep learning model for the automatic detection of AL in panoramic radiographs. The model achieved moderate to high performance levels across standard evaluation metrics, demonstrating its potential to aid in clinical assessment. Nonetheless, the inherent limitations of two-dimensional imaging should be considered, and further studies involving different imaging modalities and different deep learning architectures aimed at increasing accuracy, generalisability, and clinical relevance, are recommended.

## Figures and Tables

**Table 1:** Summary of Model Training Hyperparameters and Hardware Specifications.

Parameter	Specification
Model Architecture	YOLOv8x-seg (Extra-large Segmentation)
Hardware (GPU)	Dell PowerEdge T640 Compute Server equipped with an NVIDIA Tesla V100 GPU (16 GB VRAM)
Framework	PyTorch with CUDA Acceleration
Input Resolution	1280 × 1280 pixels
Optimizer	AdamW (Adam with Decoupled Weight Decay)
Initial Learning Rate	0.01 (with Linear Decay)
Batch Size	16
Total Epochs	700
Loss Functions	BCE (Segmentation & Classification), CloU&DFL (Localization)
Augmentation	Blur, CLAHE, Gaussian Noise
Checkpoint Selection	Best validation mAP@0.5 (Highest Mean Average Precision)
Total Training Time	Approximately 18 h

**Table 2:** Performance metrics of the YOLOv8x model for AL detection on the test dataset.

Metric	Value	95% Confidence Interval (CI)
True positives	27	-
False positives	9	-
False negatives	14	-
Precision	0.75	0.59–0.86
Recall	0.6585	0.51–0.78
F1-score	0.7013	0.58–0.80
mAP@0.5	0.739	0.59–0.85

## Data Availability

The data presented in this study are available on request from the corresponding author due to privacy and ethical restrictions.

## Abbreviations

The following abbreviations are used in this manuscript:

AL	Anterior Loop
CBCT	Cone-Beam Computed Tomography
AI	Artificial Intelligence
mAP	Mean Average Precision
IoU	Intersection Over Union
TP	True Positive
FN	False Negative
FP	False Positive
